# Supplementary figures and images for: NleB2 from enteropathogenic Escherichia coli is a novel arginine-glucose transferase effector
Source: PLoS Pathog. 2021 Jun 16;17(6):e1009658. doi: 10.1371/journal.ppat.1009658 (PMC8238200; doi:10.1371/journal.ppat.1009658)

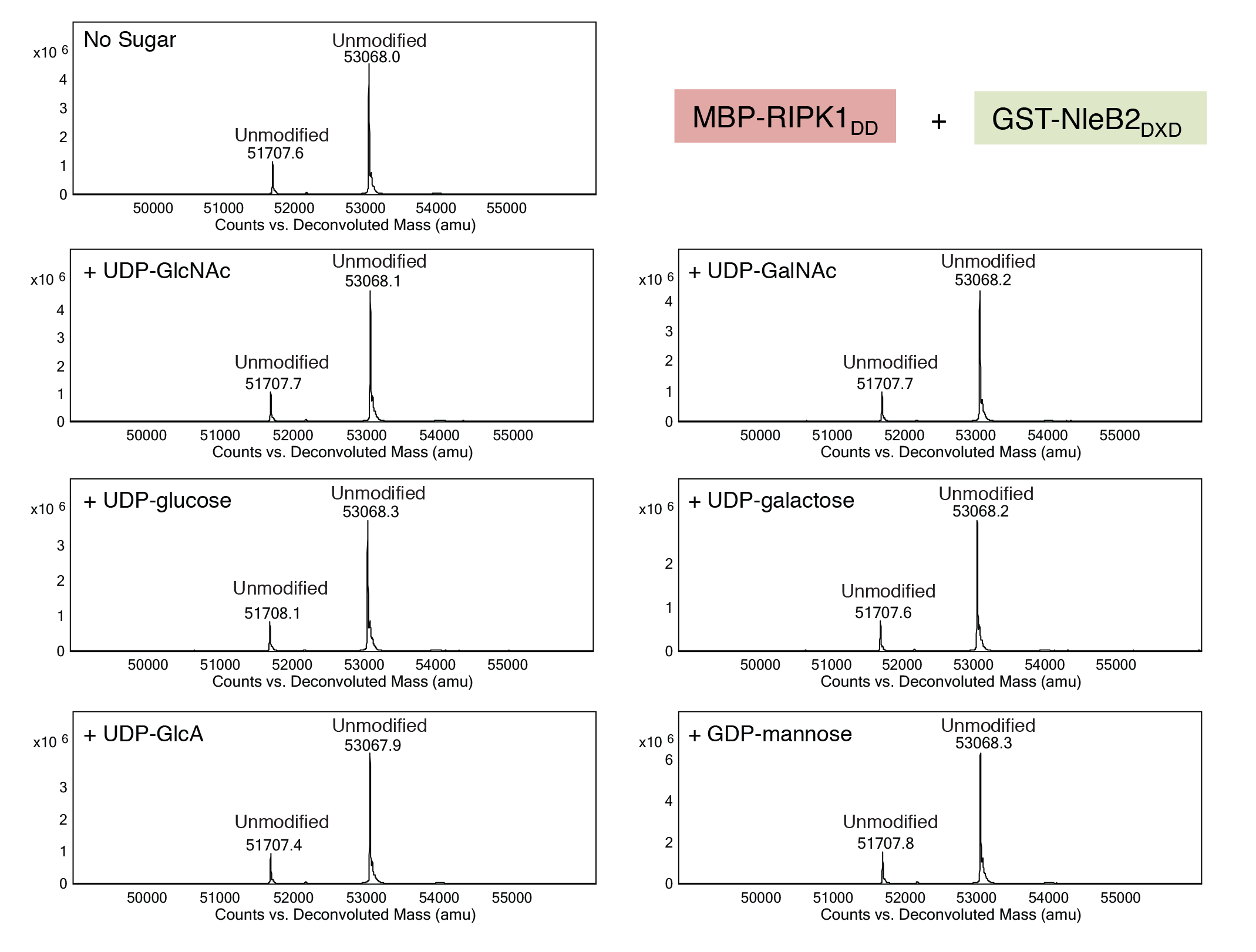

Supplement: S1 Fig — Deconvoluted intact mass spectra of MBP-RIPK1DD incubated with GST-NleB2 either without sugar donors, or in the presence of one of UDP-GlcNAc, UDP-glucose, UDP-GalNAc, UDP-galactose, UDP-glucuronic acid or GDP-mannose at 50 μM. (TIF) [file ppat.1009658.s001.tif]

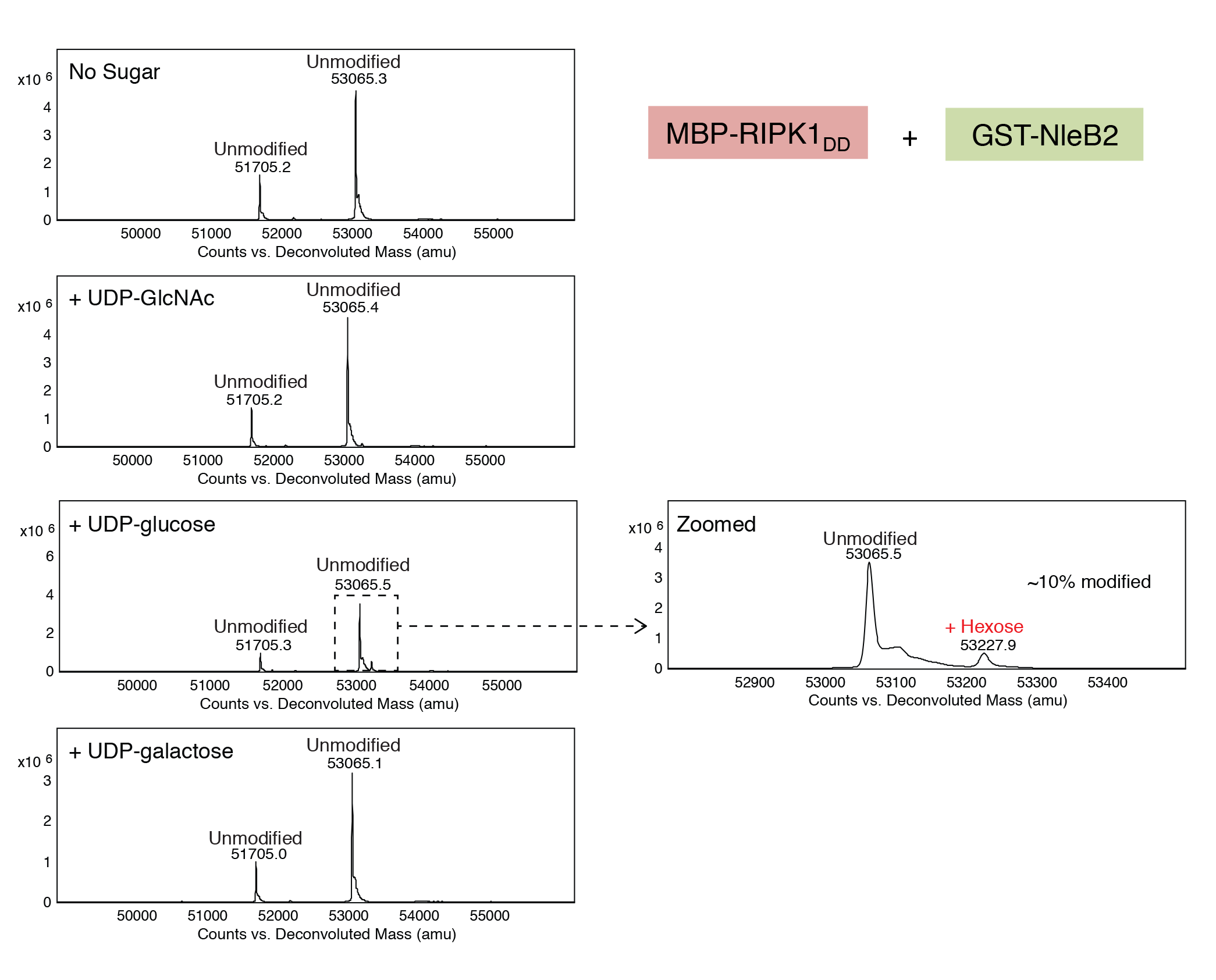

Supplement: S2 Fig — MBP-RIPK1DD was incubated with GST-NleB2 either without sugar donors, or in the presence of one of UDP-GlcNAc, UDP-glucose, or UDP-galactose at 0.5 μM for only 20 minutes. (TIF) [file ppat.1009658.s002.tif]

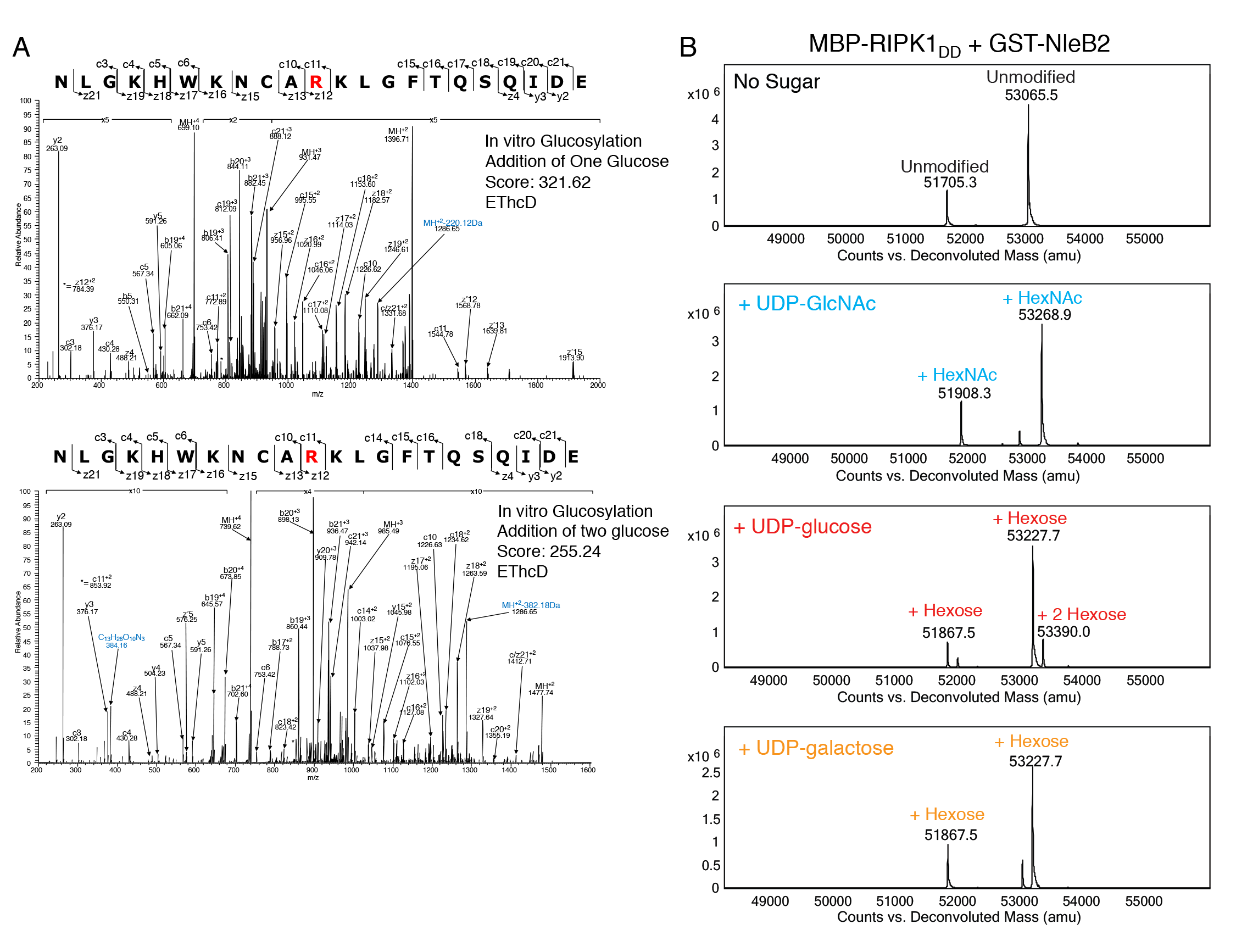

Supplement: S3 Fig — (A) Peptide isolated from MBP-RIPK1DD showing single and double Arg-glucose modifications. MBP-RIPK1DD was incubated with GST-NleB2 in the presence of 10mM UDP-glucose. (B) Intact mass spectra of MBP-RIPK1DD incubated with GST-NleB2 either without sugar donors, or in the presence of one of UDP-GlcNAc, UDP-glucose or UDP-galactose at 10 mM. (TIF) [file ppat.1009658.s003.tif]

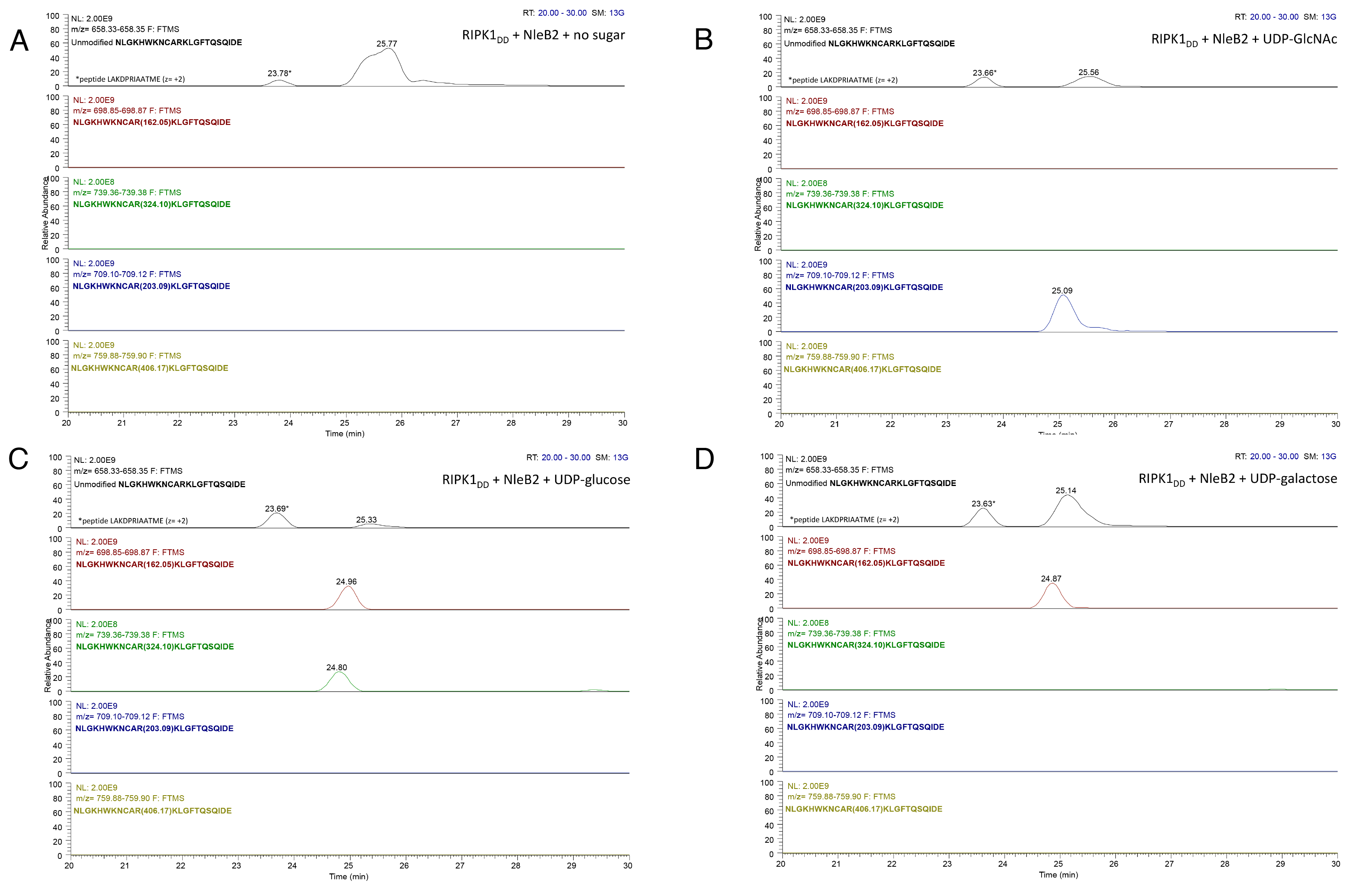

Supplement: S4 Fig — The glycosylated and non-glycosylated forms of the Arg603-containing Glu-C peptide NLGKHWKNCARKLGFTQSQIDE from MBP-RIPK1DD observed after incubation of GST-NleB2 without sugars (A), or in the presence of 10mM UDP-GlcNAc (B), UDP-glucose (C) or UDP-galactose (D) are shown. (TIF) [file ppat.1009658.s004.tif]

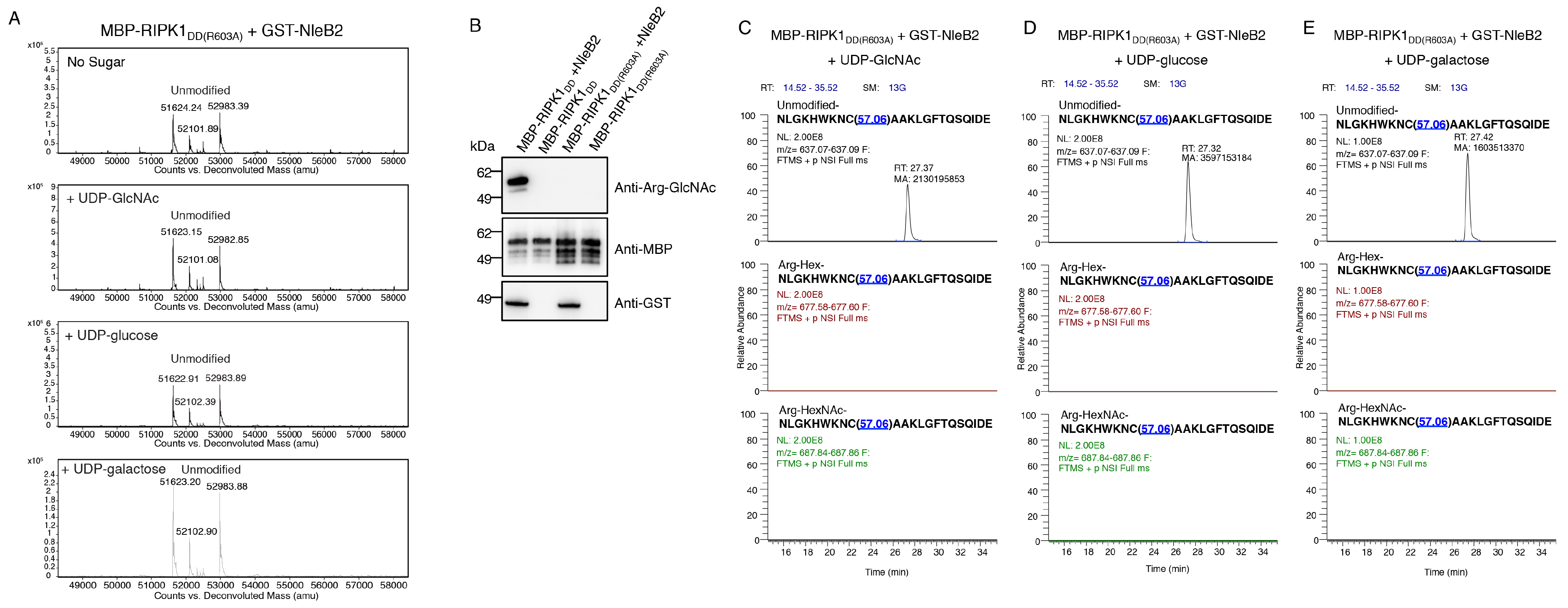

Supplement: S5 Fig — (A) Deconvoluted intact mass spectra of MBP-RIPK1DD(R603A) (Full-length expected average mass 52996 Da) incubated with GST-NleB2 either without sugar donors, or in the presence of one of UDP-GlcNAc, UDP-glucose or UDP-galactose at 10 mM. (B) Immunoblots of in vitro glycosylation assays. MBP-RIPK1DD or MBP-RIPK1DD(R503A) were incubated in the presence of 25 μM UDP-GlcNAc either alone, or with GST-NleB2. Proteins were probed with anti-ArgGlcNAc, or anti-MBP and anti-GST as controls. Representative of at least 3 experiments. (C-E) Extracted ion chromatograms of Glu-C digested MBP-RIPK1DD(R603A) after incubation with GST-NleB2 in the presence of 10mM UDP-GlcNAc (C), UDP-glucose (D) or UDP-galactose (E). (TIF) [file ppat.1009658.s005.tif]

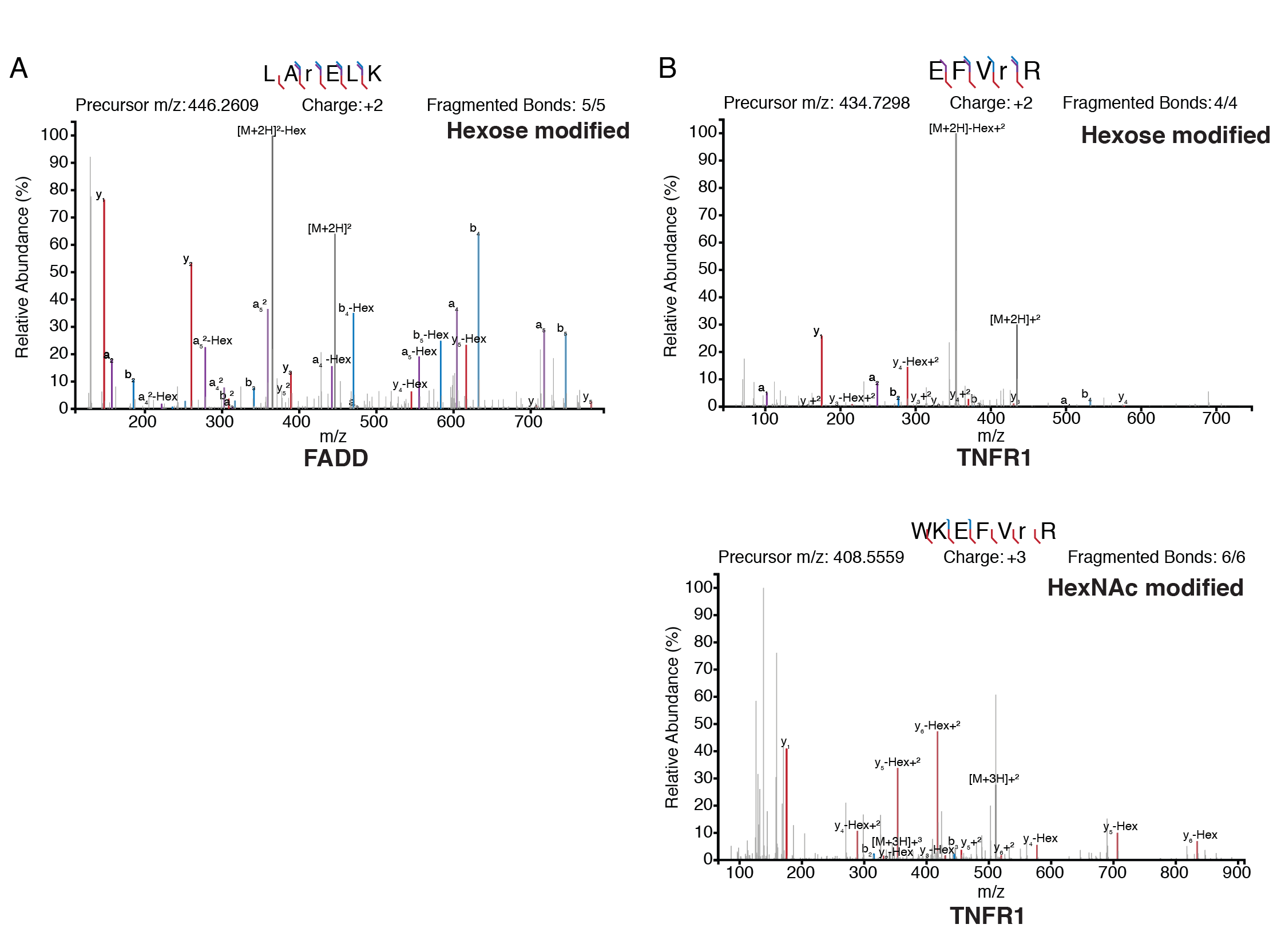

Supplement: S6 Fig — (A) Peptide isolated from His-FADD showing hexose modification of Arg117. His-FADD was incubated with GST-NleB2 in the presence of 10mM UDP-glucose in vitro. (B) Peptides isolated from Flag-TNFR1DD showing hexose and HexNAc modification of Arg376. Flag-TNFR1DD was immunoprecipitated from HEK293T cells co-expressing GFP-NleB2. (TIF) [file ppat.1009658.s006.tif]

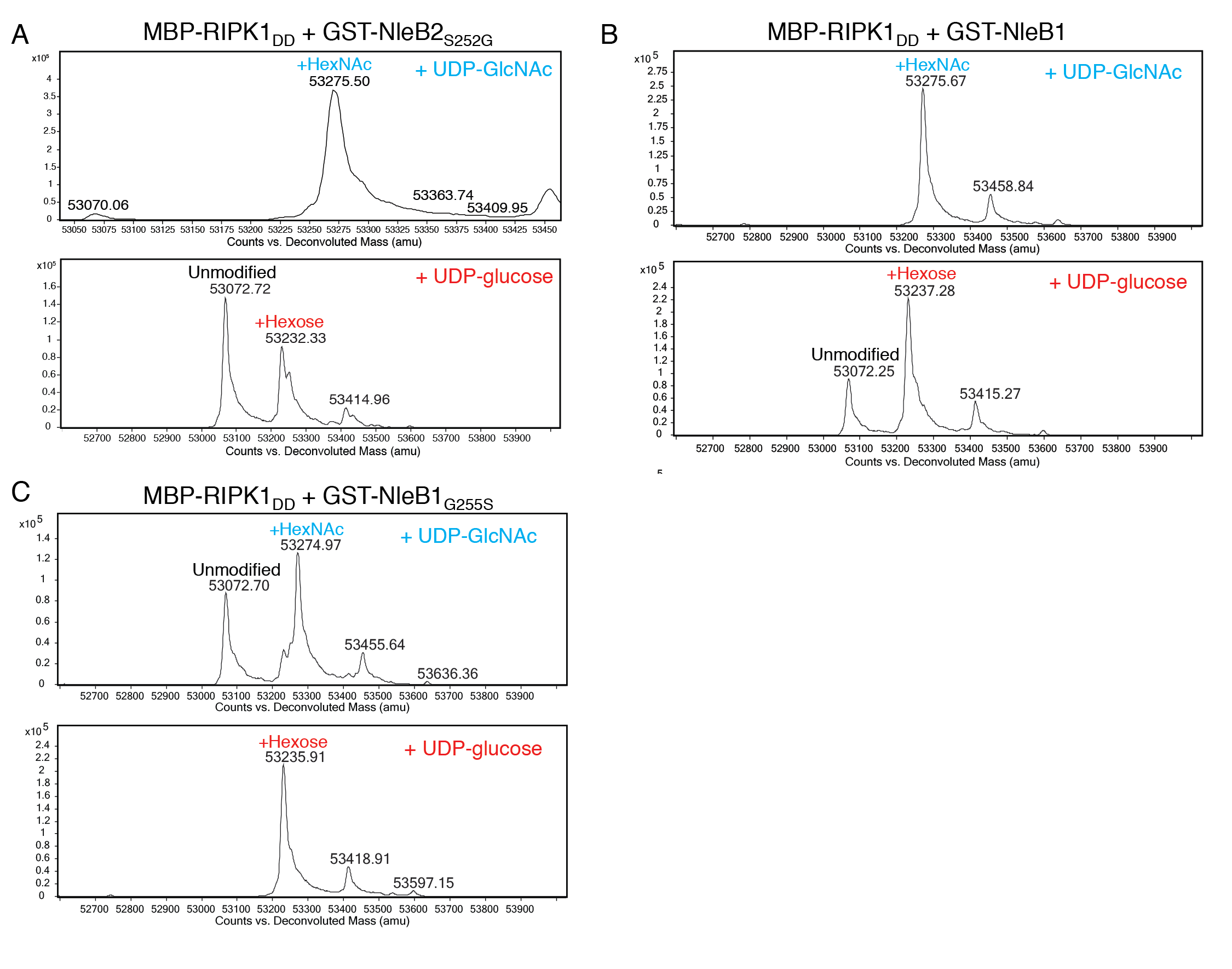

Supplement: S7 Fig — Deconvoluted intact mass spectra of MBP-RIPK1DD incubated with GST-NleB2S252G (A), GST-NleB1 (B) or GST-NleB1G255S (C) in the presence of either UDP-GlcNAc or UDP-glucose at 50 μM. (TIF) [file ppat.1009658.s007.tif]

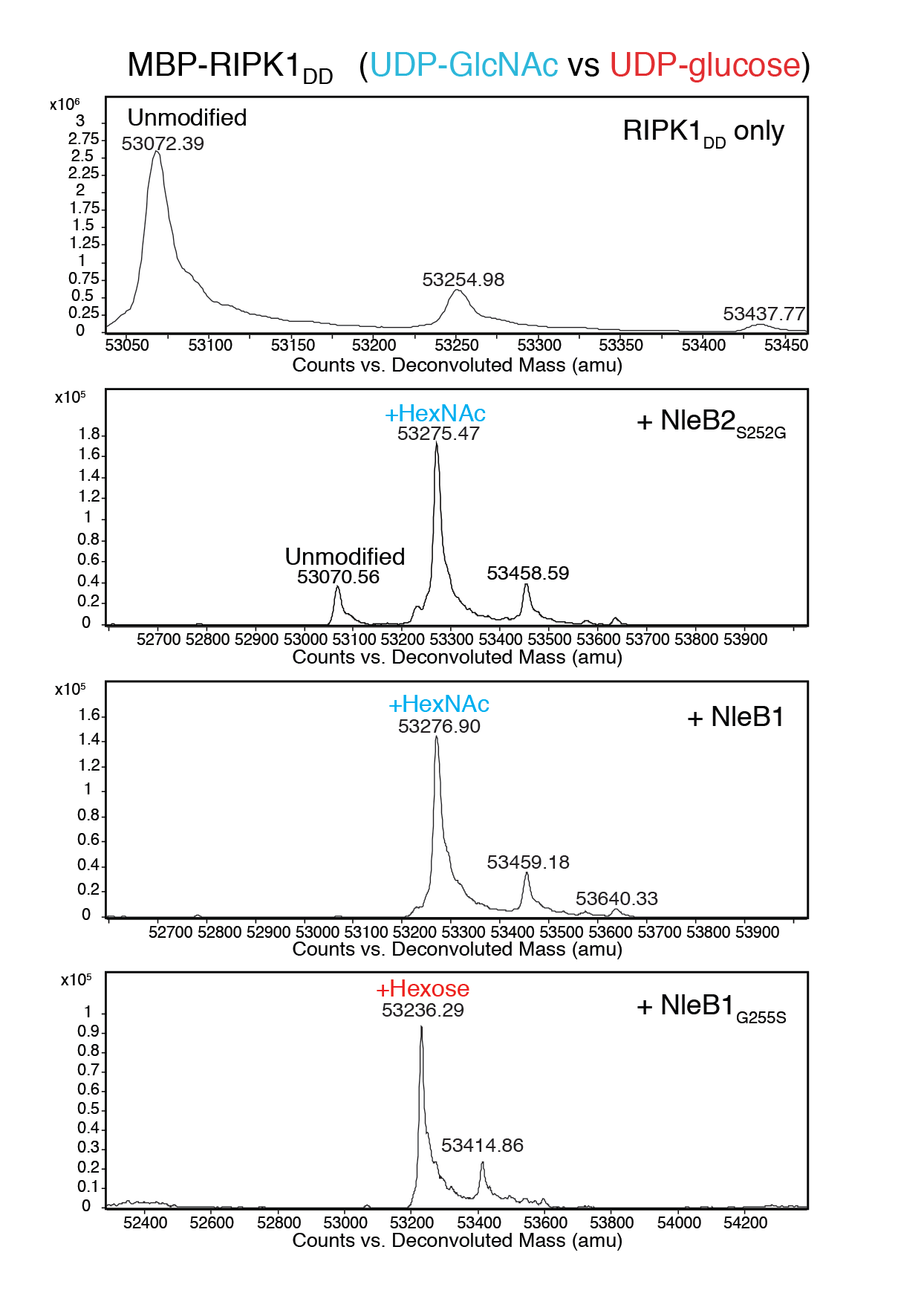

Supplement: S8 Fig — Deconvoluted intact mass spectra of in vitro sugar donor competition assays. MBP-RIPK1DD was incubated without sugar donors, or in the presence of 25 μM UDP-GlcNAc and UDP-glucose with either GST-NleB2S252G, GST-NleB1 or GST-NleB1G255S. (TIF) [file ppat.1009658.s008.tif]

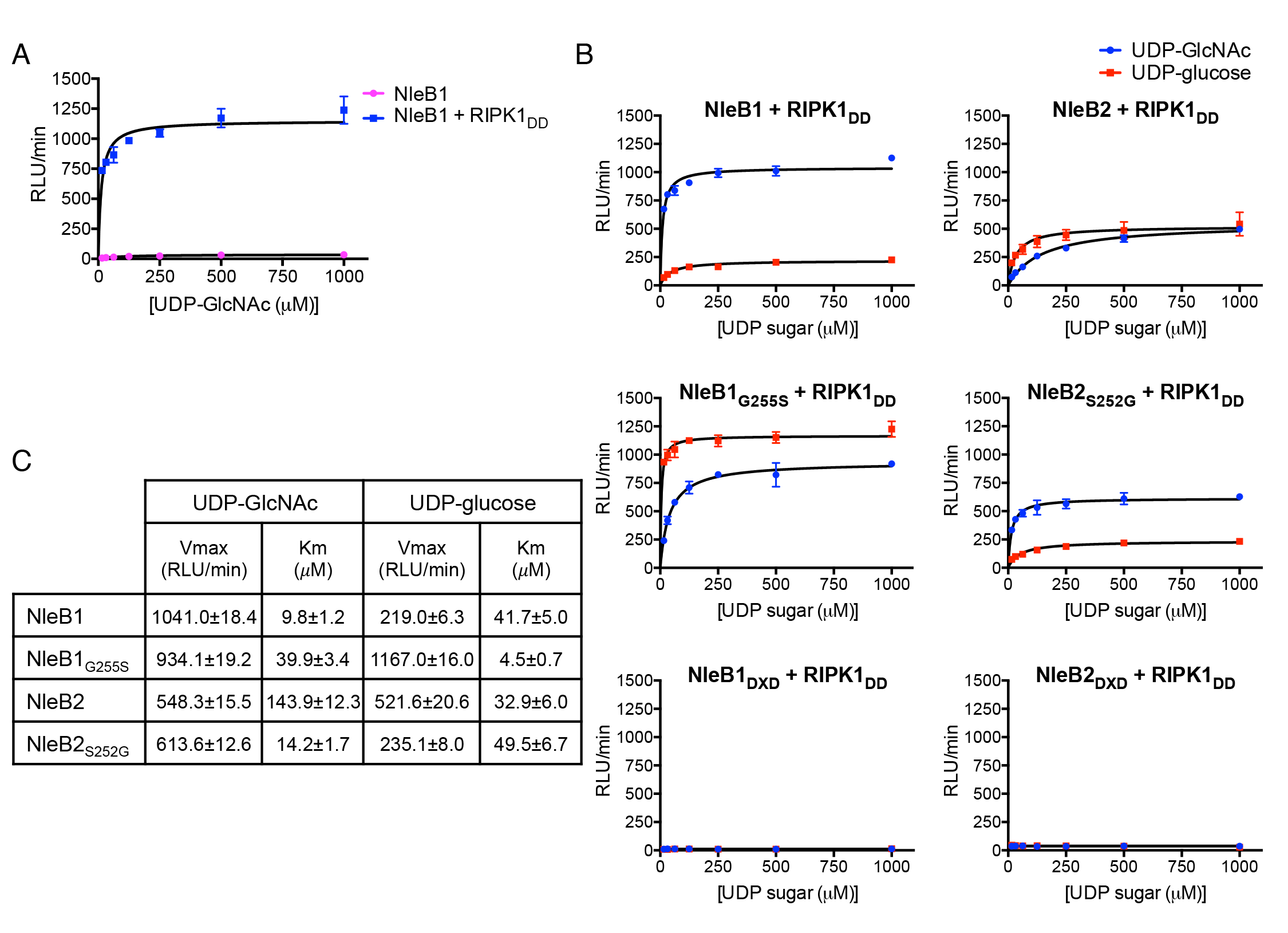

Supplement: S9 Fig — (A) Michaelis-Menten kinetics for NleB1 and UDP-GlcNAc as measured using UDP-Glo assay. UDP release was measured after a 30 minute reaction of 150 nM GST-NleB1 in the presence of titrated concentrations of UDP-GlcNAc alone, or in the presence of UDP-GlcNAc and 1 μM MBP-RIPK1. The mean relative light units (RLU) detected from two replicates is shown with error bars representing standard deviation. (B) Michaelis-Menten kinetics for NleB1, NleB2 and derivatives in the presence of UDP-GlcNAc or UDP-glucose as observed using UDP-Glo assays. UDP release was measured after a 30 minute reaction of 150 nM GST-NleB1, GST-NleB2 or derivatives in the presence of 1 μM MBP-RIPK1 and titrated concentrations of either UDP-GlcNAc or UDP-glucose. The mean relative light units (RLU) detected from three replicates is shown with error bars representing standard deviation. (C) Vmax and Km values calculated from the data in (B) using the non-linear regression fit Michaelis-Menten equation in GraphPad Prism. Values shown are ± standard deviation. (TIF) [file ppat.1009658.s009.tif]

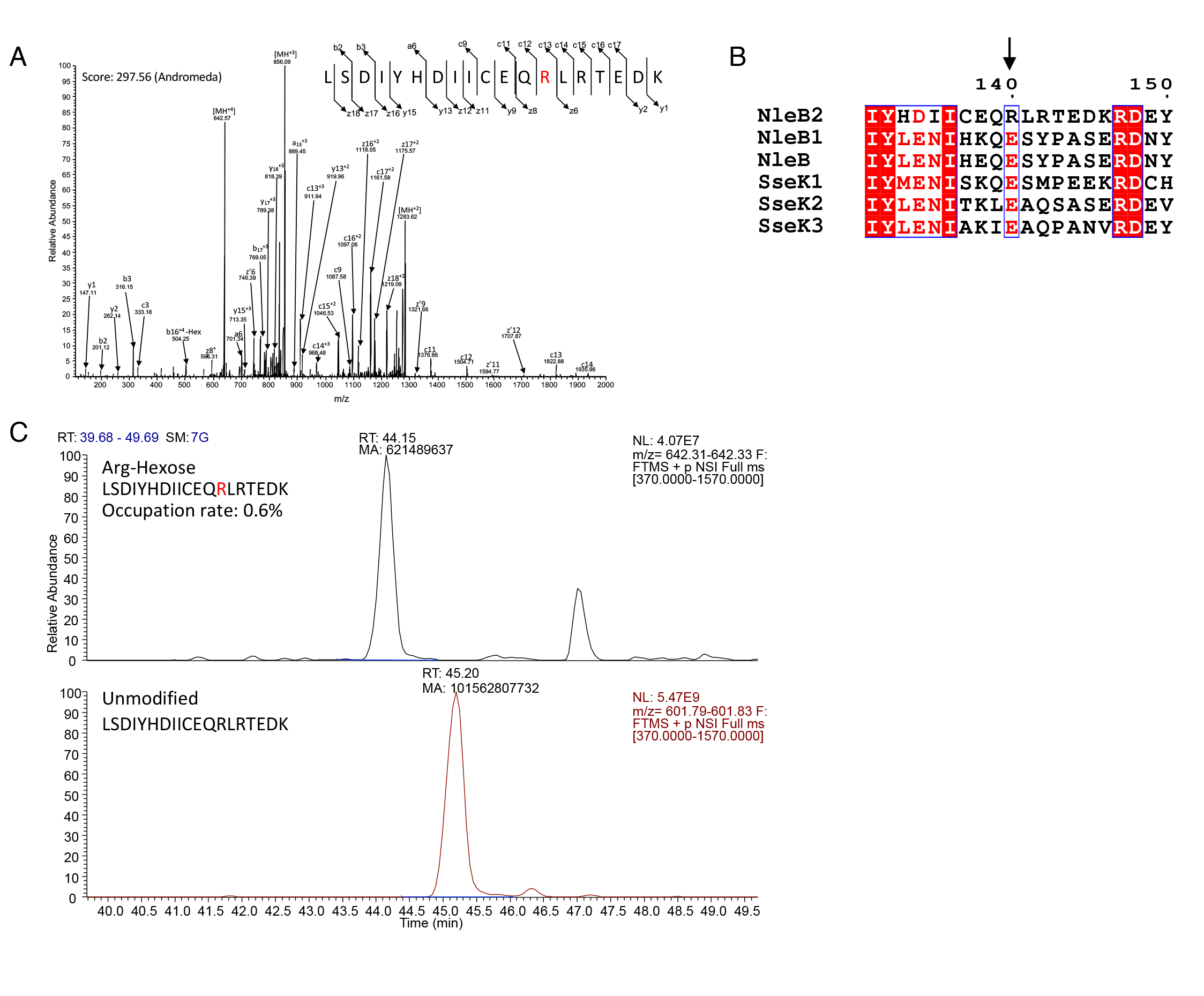

Supplement: S10 Fig — (A) Peptide isolated from Lys-C digest of GST-NleB2 showing hexose modification of Arg140 in NleB2. (B) Alignment of NleB2 and NleB1 from EPEC O127:H6 strain E2348/69, NleB from Citrobacter rodentium strain ICC168 and SseK1, SseK2 and SseK3 from Salmonella enterica serovar Typhimurium strain SL1344. Arrow indicates arginine 140 within NleB2. Alignment was performed using ClustalW and visualised using ESPript. (C) Extracted ion chromatograms of GST-NleB2 showing the glycosylated and non-glycosylated forms of the Arg140-containing Lys-C peptide LSDIYHDIICEQRLRTEDK. (TIF) [file ppat.1009658.s010.tif]
