# Supplementary material for: NleB2 from enteropathogenic Escherichia coli is a novel arginine-glucose transferase effector
Source: PLoS Pathog. 2021 Jun 16;17(6):e1009658. doi: 10.1371/journal.ppat.1009658 (PMC8238200; doi:10.1371/journal.ppat.1009658)
Supplement: Attachment — Submitted filename: Reviewer comments_NleB2.docx [file ppat.1009658.s013.docx]

*All the reviewers highly evaluated this study. Please consider the additional experiments that Reviewer 3 suggested, as the data would be great input to the story.

Reviewer Comments (if any, and for reference):

Reviewer's Responses to Questions*

***Part I - Summary*** *Please use this section to discuss strengths/weaknesses of study, novelty/significance, general execution and scholarship.*

*Reviewer #1: The manuscript is interesting and reader friendly, as it’s easy to follow. Very good article overall. Note that I am not qualified to evaluate the quality of the MS analysis, but I assume that it is OK. A major question that remain open is related to the function of native NleB2 in the context of infection. What is the phenotype of NleB2 mutant, if any? Is this phenotype, or lack of it, related simply to the native expression levels? I hope that the authors can provide answers and if not they should discuss this issue. Also. it would be nice, although not essential, to have the Km towards the different substrates of NleB1, NleB2, NleB1 G255S and NleB2 S252G. Finally, Figure 7B, the level of NleB1G255S is high* comparing to pNleB1. Please comment on this. Is this a stability issue?

Thank you for the positive review of our manuscript.

We have included a discussion of the *nleB2* mutant phenotype and NleB2 expression and translocation levels compared to NleB1 **(Lines 420-435)**

Km values have been calculated for NleB1, NleB1_G255S_, NleB2, NleB2_S252G_ **(Supp Fig. 9, Lines 261-282)**

The reason for higher levels of NleB1_G255S_ detected compared to NleB1 in complementation strains is unclear. We believe that the mutation may improve stability of the protein, or enable it to be more readily detected by the polyclonal NleB1 antibody but this is unclear at this stage.

*Reviewer #2: In this paper, Giogha and colleagues have convincingly characterized a novel bacterial arginine-glucose transferase. Additional biochemical analysis, including kinetic analysis of this enzyme for sugar preferences and usages, is anticipated to provide other critical information. Furthermore, a molecular tool specifically detecting host glucosylated substrates is also expected to help identify this enzyme's host substrates (if there are more) and characterize the roles of this effector in the context of infection. Nonetheless, the data presented in this paper sufficiently and convincingly support the finding of a new arginine-glucose transferase enzyme. My comments and suggestions are as follows.*

Thank you for the detailed and helpful suggestions. We have performed kinetic analysis of each of the enzymes for UDP-GlcNAc and UDP-glucose as suggested **(Supp Fig. 9, Lines 261-282).**

*1. Is the temporal expression regulation of NleB1 and B2 during infection known? Are both effectors expressed and translocated to host cells at a similar level during the course of infection? Or Do those differ in different conditions? Relevant information can be discussed in lines 327-338. If appropriate, this discussion can be extended by comparing and contrasting with SseK1, K2, and K3’s expressions, translocations, and roles.*

We have added a discussion about the levels of NleB2 and NleB1 translocation to our manuscript. We did not comment on SseK1-3, as they are less relevant to the study. (**Lines 420-435)**

*2. In lines 229-232, the band of approximately 28 kDa is pointed out. What is your interpretation? Is this an auto-glycosylated band? It seems that the authors think this is an important observation, but a relevant description is omitted in the paper.*

We propose that this band may be FADD, given the strong preference of NleB1 for FADD (Scott *et al* JBC 2017), and have updated the manuscript accordingly **(Lines 248-250).**

*3. In lines 249-251, this is a confusing sentence. This is associated with many bands in the blot shown in Fig 7B. Please consider rephrasing the sentence and/or improving the figure panel and legend.*

We apologise for the lack of clarity and have simplified the sentence in our revised manuscript. (**Lines 290-292)**

*4. Figure legends need to be extended to explain figure panels better. For instance, Fig. 1A acronym DDO and QDO are used without full names. In Fig. 1B, TNF-a needs to be used instead of TNF since there are different forms. The acronym SEM also needs to be spelled out in Fig. 1.*

Thank you for the suggestion, we have made the following changes:

The DDO and QDO acronyms in Figure 1 have been explained in full, and the acronym SEM has been explained in full for Figure 1 and Figure 7.

The first instance of PAGE in Figure 2 has also been corrected and explained in full.

We have left Figure 1B unmodified, as the current convention is to use TNF rather than TNFa (JAMA Dermatol. 2016;152(5):557)

*5. Fig. 5B legend does not describe color codes of the sugar moiety; for instance, indicating color codes for nitrogen (blue) and oxygen (red) will help orient the N-acetyl group's location.*

We have updated the figure legend to help readers interpret the figure as suggested.

*6. Fig. 6A and 6B descriptions are the same. Exposure time difference should be indicated in Fig. 6B legend and lines 229-232.*

Apologies, the difference between the samples was that a more sensitive chemiluminescent substrate was used for 6B. The main text, figure legend and methods have been updated to reflect this. **(Lines 247-248)**

*7. The authors can consider showing individual data points in bar graphs (e.g., Fig. 1B, 7A).*

Individual data points are now showed alongside bar graphs for Fig 1B and 7A.

*8. Supplementary figure panels S4A, B, C, & D, S5C, D, & E, and S8C are cropped, and no graphs are shown.*

Apologies for the error which occurred upon creation of the pdf file, this has now been corrected.

*9. At line 183, please confirm whether 10 mM UPD-sugar is correct since, in other experiments, 50 µM or 25 µM sugars are indicated (e.g., Figure legends to Figs. 3-6).*

This is correct, for this particular assay we used a higher concentration of UDP-sugar to promote complete modification of RIPK1. The manuscript has been updated to reflect this explanation. **(Lines 183-184)**

*Reviewer #3: In this paper, Giogha et. al. present evidence that the T3SS effector NleB2 from EPEC and EHEC transfers glucose from UDP-glucose to Arg residues on the Death Domain (DD) of RIPK1. Previous studies have shown that the NleB2 paralog, NleB1 transfers GlcNAc from UDP-GlcNAc to Arg residues on DD containing proteins involved in the host immune response. Moreover, NleB2 was tested in these studies and shown to have low levels of Arg-GlcNAcylation of TRADD. Here the authors identified an interaction between NleB2 and the DD domains of RIPK1 and TNFR1 using a yeast 2 hybrid screen and convincingly show that NleB2 (but not a catalytically inactive mutant), like NleB1, inhibits TNFa-induced NFkB activation. Global Arg-GlcNAcylation was observed in lysates from cells overexpressing NleB1 using an anti-Arg GlcNAcylation antibody but not in cells overexpressing NleB2. Infection experiments suggest that NleB2 is not involved in Arg-GlcNAcylation of host proteins during EPEC infection. The authors go on to show that NleB2 utilizes UDP-glucose to Arg-glucosylate RIPK1 on Arg603 within the DD of RIPK1. Structure guided mutagenesis of NleB2 based on the active site architecture of an NleB homolog, revealed that Ser 252 in NleB2 dictates specificity between NleB1 and NleB2 for UDP-GlcNAc and UDP glucose, respectively.

Overall, this is a nice study that adds to our understanding of Arg-glucosylation and EPEC/EHEC pathogenesis. I recommend publication provided the authors can address the following points.*

***Part II – Major Issues: Key Experiments Required for Acceptance*** *Please use this section to detail the key new experiments or modifications of existing experiments that should be absolutely required to validate study conclusions.

Generally, there should be no more than 3 such required experiments or major modifications for a "Major Revision" recommendation. If more than 3 experiments are necessary to validate the study conclusions, then you are encouraged to recommend "Reject".*

*Reviewer #1: none*

*Reviewer #2: NA*

*Reviewer #3: 1. Proper kinetic measurements should really be done to compare the Km for UDP-glucose vs UDP-GlcNAc (and the other UDP-sugars used in the paper).*

Thank you for suggesting these critical experiments that have added value to our manuscript. Kinetic measurements have been performed using a UDP Glo assay **(Supp Fig. 9, Lines 261-282)**. We were unable to examine the Km of UDP-galactose due to instability of the sugar in our assays but we did compare UDP-GlcNAc and UDP-glucose usage by NleB1, NleB2 and associated site-directed mutants *in vitro*.

*2. It should be shown that NleB2 mediates glucosylation of proteins during infection. If one were to IP overexpressed RIPK1 from infected cells, do you observe Arg-glucosylation by MS? Does this go away in NleB2 KO cells?*

Thank you for the suggestion, we have completed the experiment as requested **(Fig. 8, Supp Table 2)**. We did indeed identify arginine-hexose modification of overexpressed RIPK1 during EPEC infection that was dependent on NleB2. **(Lines 294-321)**

*3. The authors should test whether other potential DD-containing substrates are glucosylated by NleB2. Eg. TNFR1, TRADD, etc.*

We tested whether FADD, or the death domains of FAS and TRAILR2 were glucosylated by NleB2 *in vitro* and, due to the lack of solubility of TRADD and the death domain of TNFR1, we examined modification of these substrates in HEK293T cells co-expressing NleB2 and TRADD or TNFR1 **(Supp Fig. 6, Supp Table 1, Lines 193-204)**

***Part III – Minor Issues: Editorial and Data Presentation Modifications*** *Please use this section for editorial suggestions as well as relatively minor modifications of existing data that would enhance clarity.*

*Reviewer #1: in the summary*

*Reviewer #2: Minor issues are indicated above.*

*Reviewer #3: (No Response)*
